# Antibiotic susceptibility and genetic relatedness of *Shigella* species isolated from food and human stool samples in Qazvin, Iran

**DOI:** 10.1186/s13104-021-05554-3

**Published:** 2021-04-17

**Authors:** Babak Pakbin, Abdollah Didban, Yousef Khazaye Monfared, Razzagh Mahmoudi, Amir Peymani, Mohammad Reza Modabber

**Affiliations:** 1 Children Growth Research Center, Research Institute for Prevention of Non-Communicable Diseases, Qazvin University of Medical Sciences, Bahonar Blvd., P.O. Box: 34185-754, Qazvin, Iran; 2 M.Sc. of Medical-Biotechnology, Dezful University of Medical Sciences, Dezful, Iran; 3 Medical Microbiology Research Center, Qazvin University of Medical Sciences, Qazvin, Iran

**Keywords:** *Shigella* species, Genetic relatedness, Antimicrobial susceptibility, Food samples, Stool specimens

## Abstract

**Objective:**

The aim of this study was to investigate the genetic relatedness and antimicrobial resistance among *Shigella* species isolated from food and stool samples. Using cross sectional study method, *Shigella* spp. were isolated from food and clinical samples using culture-based, biochemical and serological methods. Antimicrobial susceptibility and genetic relatedness among the isolates were evaluated using disk diffusion and RAPD-PCR methods respectively.

**Results:**

The prevalence of *Shigella* spp. were 4.84 and 7.7% in food and stool samples respectively. All food isolates were *Sh. sonnei*. 91.42% of the *Shigella* stool isolates were *Sh. sonnei*. 62.5% of food isolates were resistant to tetracycline. 46.8, 50 and 65.8% of clinical isolates were resistant to imipenem, amikacin and azithromycin respectively. 50 and 85.7% of the food and clinical isolates respectively were MDR. Dendrogram generated by RAPD-PCR showed that the isolates from food and stool samples were categorized in a same group. Close genetic relatedness between MDR Shigella isolates from food and clinical samples indicate that foods can be considered as one of the main vehicles for transmission of MDR *Shigella* to human causing acute diseases. Survey of MDR *Shigella* among food and clinical samples is strongly suggested to be implemented.

## Introduction

*Shigella* are non-motile, gram-negative and rod-shaped bacteria belonging to the *Enterobacteriacae* family. During an epidemic outbreak, in 1898, *Shigella* was isolated for the first time from bacillary dysentery cases by Kiyoshi Shiga in Japan [1]. *Shigella* comprises four species including *Sh. dysenteriae, Sh. flexneri, Sh. boydii* and *Sh. sonnei* [2]. *Shigella* spp. contribute to intestinal disorders including mild to severe bloody diarrhea in children and adults [3]. However*, Sh. dysenteriae* type 1 cause hemorrhagic uremic syndrome (HUS) as an extraintestinal pathogen via releasing shiga-toxins [4]. Prevalence of *Shigella* spp. is geographically different, as *Sh. sonnei* and *Sh. flexneri* have more been isolated from patients in industrialized and developing countries respectively. Shigellosis have commonly been seen among the children under the age of 5 years old especially in low and middle-income countries. *Shigella* is an important foodborne pathogen and sometimes is transmitted via animals. Regarding the remarkable reduction in mortality of this pathogen in the recent decades, there are still more than 160,000 deaths annually caused by *Shigella* worldwide [5]. Drug resistance of human pathogens is one of the main concerns of public health. Multidrug resistant (MDR) is defined when a pathogen is resistant to two or more antibiotics from different groups. Several studies have been highlighting the importance of MDR human and animal pathogens [6–11]. Spread of MDR Shigella is associated with severe outcomes and leads to failure in treatment. Foods have been considered as an important vehicle for transmission of pathogens. A few studies demonstrated a close genetic relatedness between the *Shigella* isolates from food and clinical samples indicating that *Shigella* may be transmitted through food to human leading to intestinal and extraintestinal infectious diseases [12, 13].

Random amplified polymorphic DNA (RAPD) method has widely been used to investigate the genetic relatedness between the pathogenic isolates from different sources [14]. Many researchers used RAPD-PCR to study the genetic diversity among pathogenic isolates [15]. Several studies have been performed to characterize the antibiotic resistance in MDR *Shigella* isolates from food and clinical samples [13]; however, there are limited literature available that provides the genetic relatedness among MDR *Shigella* isolates from food and clinical samples. The objective of the present study was to investigate the genetic relatedness among the MDR *Shigella* spp. isolated from food samples and stool specimens from the patients with diarrhea.

## Main text

### Bacterial isolates

453 children between the age of 2–5 years old, including 242 males and 211 females, who visited the department of children health and diseases (Qazvin children hospital, Qazvin, Iran) for diarrhea and dysentery from November 2019 to July 2020 were enrolled into the study and forwarded to the clinical central Lab of Qazvin children hospital, Qazvin, Iran. All stool specimens were collected into clean, sterile and disposal containers according to the protocol described by WHO Laboratory investigations, manual of Enteric infections for fecal sample preparation [11]. Totally 165 food samples, consisting of 55 samples of each food item, including minced meat (100 g), vegetable salad (500 g) and raw milk (1 L) were collected from local stores, located in different areas of Qazvin city, from September to December 2019. All clinical and food samples were kept at 4 °C prior to bacterial isolation and were immediately transported to the central Lab. *S. sonnei* ATCC 25,931; *S. boydii* ATCC 12,030; and *S. flexneri* ATCC 12,022 were purchased from Pasteur institute of Iran (Pasteur institute, Tehran, Iran) and used as the reference strains in this study. All reference strains were activated by inoculation into Bovine Heart Infusion (BHI) broth and incubation at 37 °C for 24 h.

### *Shigella* spp. isolation and identification

After homogenization, stool samples were directly streaked onto MacConkey and Salmonella-Shigella (SS) agar (Promedia, Spain) and incubated anaerobically at 37 °C for 24 h. H_2_S negative and non-lactose fermentative colonies were selected for further biochemical tests. Isolation of *Shigella* spp. from food samples were conducted using the method described by Mokhtari et al. [13]. First, 25 g or mL of each food sample were homogenized using Stomacher BagMixer Lab blender (InterScience, France) for 1 min. Homogenized samples were mixed and diluted with 225 mL of buffered peptone water broth (BPW, Promedia, Spain) and incubated for 24 h at 37 °C. Diluted samples were inoculated into Shigella-broth (Promedia, Spain) and incubated at 37 °C overnight. Enriched samples were streaked onto the MacConkey and SS agar (Promedia, Spain) plates and incubated anaerobically at 37 °C for 24 h. Urease, motility, triple sugar iron, lysin iron decarboxylase and IMViC tests were performed for biochemical confirmation of the presumptive colonies. Serological assay was used for identification of *Shigella* genus using Difco Antisera Kit (BD-Difco Co. USA). Different species of *Shigella* isolates were also identified by serological tests using *Shigella* species Difco Antisera Kit (BD-Difco Co. USA). All serological tests were conducted according to the kit manufacturers` instruction [12].

### Antimicrobial Susceptibility testing

The antimicrobial susceptibility of the isolates was evaluated using the disk diffusion technique which was previously described by Marami et al. [16] in accordance with the guideline of Clinical and Laboratory Standards Institute (CLSI) [17]. Seven antimicrobial disks (Oxoid, Ltd, UK) including chloramphenicol (30 mg), tetracyclin (30 mg), ampicillin (10 mg), amikacin (30 mg), imipenem (10 mg), azithromycin (15 mg) and nalidixic acid (30 mg) were applied. When the isolates were resistant to two or more different antimicrobial agents, multidrug resistance (MDR) was defined.

### DNA extraction

All strains were inoculated into trypticase soy broth (TSB, Promedia, Spain), incubated at 37 °C overnight and centrifuged at 3500 rpm for 10 min. After discarding the supernatant, precipitated cell pellets were subjected to DNA extraction using the gram-negative bacterial DNA extraction kit (Sinaclon, Iran) according to the manufacturers` instruction. Quantity and quality of the extracted genome were measured using NanoDrop Spectrophotometer (Thermo Scientific, USA). Concentration of the DNA templates were adjusted to 50 ng. µL^−1^.

### RAPD-PCR based genotyping and genomic fingerprinting

In this study, primer UBC245: 5ʹ- CGC GTG CCA G-3 ʹ [18], previously used for RAPD-genotyping of *Shigella*, was used. Each reaction tube contained 25 µL of total reaction volume including 12.5 µL of PCR 2X master mix kit (Ampliqon, Denmark), 0.5 µL of primer (50 µM), 1 µL of DNA template (50 ng. µL^−1^) and deionized sterilized water to the final reaction volume. Thermal cycling was run using Bio-rad T100 machine (Bio-rad, USA) in the following program: initial denaturation 5 min at 95 °C; 35 cycles of 95 °C for 1 min, 36 °C for 1 min, 72 °C for 5 min; and 5 min at 72 °C as the final extension. PCR products were characterized using gel electrophoresis for 2 h at 70 v on 1.5% w/v agarose (Merck, Germany) containing DNA safe stain (CinnaGen, Iran) along with 100-bp DNA ladder. Fluorescent banding patterns were recorded using gel documentation system (Mahazma, Iran). For genomic fingerprinting, DNA banding patterns were interpreted using PyElph software version 1.4 [19]. Similarities were calculated and the unweighted pair group method with arithmetic mean (UPGMA) dendrogram was drawn regarding Dice coefficient by NTSYSpc software version 2.20 (Applied Biostatics Inc., USA).

## Results

**Fig. 1:**
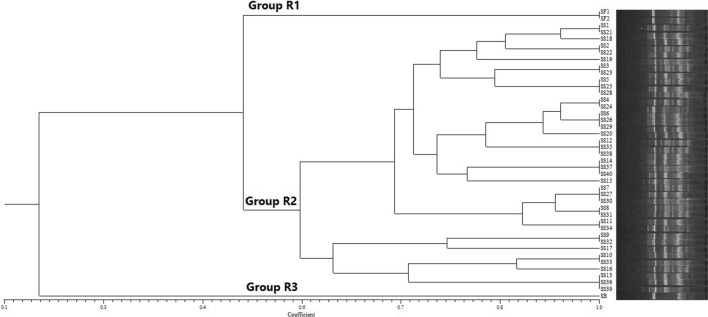
Dendrogram generated by RAPD-PCR banding pattern of 43 different *Shigella* spp. isolated from food and clinical samples including 3 significant clusters (groups R1, R2 and R3)

Shigella were detected in 8 food samples (4.84%). All of these isolates were *Sh. sonnei* and isolated from vegetable salad samples. *Shigella* spp. were detected in 35 stool samples (7.7%) including 32 *Sh. sonnei* (91.42%), 2 *Sh. flexneri* (5.71%) and 1 *Sh. boydii* (2.85%) isolate. We have not isolated any *Sh. dysenteriae*. As shown in Table 1, 62.5% of food isolates were resistant to tetracycline and all isolates were sensitive to amikacin. 46.8, 50 and 65.8% of clinical isolates were resistant to imipenem, amikacin and azithromycin respectively. Also, all clinical isolates were sensitive to tetracycline, chloramphenicol and nalidixic acid. 4 (50%) and 30 (85.7%) of the food and clinical isolates respectively were MDR. 16 distinct amplicons ranged in size from 60 to 1100 bp were detected by RAPD-PCR formed 22 different RAPD profiles and 3 main genotypic clusters consisting of groups R1, R2 and R3 (Fig. 1). Genotypic categorization and serogroups of the isolates were demonstrated in Table 2. All *Sh. sonnei* isolated from food and stool samples were categorized together in a same cluster (Group R2) with a close genetic relatedness.

**Table 1:** Antimicrobial resistance properties of *Shigella* spp. isolated from food and stool samples

Antimicrobial agent	n (%)			
	Food samples	Stool samples		
	*S. sonnei* (n = 8)	*S. sonnei* (n = 32)	*S. flexneri* (n = 2)	*S. boydii* (n = 1)
Imipenem	1 (12.5)	11 (46.8)	1 (50)	1 (100)
Ampicillin	3 (37.5)	32 (100)	2 (100)	1 (100)
Tetracycline	5 (62.5)	0 (0)	0 (0)	0 (0)
Amikacin	0 (0)	16 (50)	1 (50)	1 (100)
Chloramphenicol	3 (37.5)	0 (0)	0 (0)	0 (0)
Nalidixic Acid	1 (12.5)	0 (0)	0 (0)	0 (0)
Azithromycin	3 (37.5)	21 (65.6)	1 (50)	1 (100)

**Table 2:** Genotypic properties and serogroup of Shigella spp. isolated from food and stool samples

ID	Species	Serogroup	Source	RAPD type
SS1	*S. sonnei*	D	Food	R2
SS2	*S. sonnei*	D	Food	R2
SS3	*S. sonnei*	D	Food	R2
SS4	*S. sonnei*	D	Food	R2
SS5	*S. sonnei*	D	Food	R2
SS6	*S. sonnei*	D	Food	R2
SS7	*S. sonnei*	D	Food	R2
SS8	*S. sonnei*	D	Food	R2
SS9	*S. sonnei*	D	Stool	R2
SS10	*S. sonnei*	D	Stool	R2
SS11	*S. sonnei*	D	Stool	R2
SS12	*S. sonnei*	D	Stool	R2
SS13	*S. sonnei*	D	Stool	R2
SS14	*S. sonnei*	D	Stool	R2
SS15	*S. sonnei*	D	Stool	R2
SS16	*S. sonnei*	D	Stool	R2
SS17	*S. sonnei*	D	Stool	R2
SS18	*S. sonnei*	D	Stool	R2
SS19	*S. sonnei*	D	Stool	R2
SS20	*S. sonnei*	D	Stool	R2
SS21	*S. sonnei*	D	Stool	R2
SS22	*S. sonnei*	D	Stool	R2
SS23	*S. sonnei*	D	Stool	R2
SS24	*S. sonnei*	D	Stool	R2
SS25	*S. sonnei*	D	Stool	R2
SS26	*S. sonnei*	D	Stool	R2
SS27	*S. sonnei*	D	Stool	R2
SS28	*S. sonnei*	D	Stool	R2
SS29	*S. sonnei*	D	Stool	R2
SS30	*S. sonnei*	D	Stool	R2
SS31	*S. sonnei*	D	Stool	R2
SS32	*S. sonnei*	D	Stool	R2
SS33	*S. sonnei*	D	Stool	R2
SS34	*S. sonnei*	D	Stool	R2
SS35	*S. sonnei*	D	Stool	R2
SS36	*S. sonnei*	D	Stool	R2
SS37	*S. sonnei*	D	Stool	R2
SS38	*S. sonnei*	D	Stool	R2
SS39	*S. sonnei*	D	Stool	R2
SS40	*S. sonnei*	D	Stool	R2
SF1	*S. flexneri*	B	Stool	R1
SF2	*S. flexneri*	B	Stool	R1
SB	*S. boydii*	C	Stool	R3

## Discussion

Shigellosis, specially caused by MDR *Shigella* spp., is now considered as one of the main public health concerns. The prevalence of this pathogen is continually increasing specially in developing countries [20]. *Shigella* spp. lead to death in children under the age of 5 years old [12]. In addition to person-to-person transmission, *Shigella* spp. are transmitted via food as a foodborne pathogen to infect human [21]. At the present study, we found higher prevalence rates of MDR *Shigella* isolates with close genetic relatedness in food and stool samples indicating poor hygiene practices and concern to public health.

The overall prevalence of the *Shigella* spp. in food and stool samples were 7.7% and 4.84% respectively. The finding of current study is significantly high compared to previous reports. Bakhshi et al. found *Shigella* spp. in 1.32% of all stool samples, collected from patients with diarrhea in Iran, consisting of *Sh. sonnei* in 92% of all isolates [22]. *Sh. sonnei* have predominantly been isolated in developed countries [23]. We also found *Sh. sonnei* predominantly in 100 and 91.42% of all isolates from food and clinical samples respectively. These findings are comparable to studies conducted before. Ranjbar et al., Nikfar et al., Zamanlou et al. and Abbasi et al. also found *Sh. sonnei* as the predominant species among the food and clinical *Shigella* isolates in Iran [24–27] indicating a recent significant change in predominant *Shigella* spp. from *flexneri* to *sonnei*.

Analysis of antimicrobial susceptibility in this study showed that the food isolates were highly resistant to tetracycline and the clinical isolates were strongly resistant to imipenem, amikacin and azithromycin. Higher susceptibility to tetracycline, chloramphenicol and nalidixic acid was also observed in clinical isolates. Shahin et al. isolated *Shigella* spp. from water samples strongly resistance to nalidixic acid and susceptible to chloramphenicol [28]. Marami et al. isolated MDR *Shigella* spp. with 75% resistance to tetracycline [16]. Aklilu et al. observed MDR *Shigella* isolates completely susceptible to amikacin. These differences in antimicrobial susceptibility patterns may be explained by different availability of antibiotics worldwide. We found different antibiotic resistance patterns between food and clinical isolates [29]. Mokhtari et al. also found significantly different antimicrobial susceptibility patterns of *Shigella* isolates obtained from food and clinical samples [13].

There are only few studies regarding the genetic relatedness among *Shigella* isolates obtained from food and clinical samples. We found a close genetic relatedness between the food and clinical isolates as they were clustered in a same group. Mokhtari et al. also demonstrated a clonal relationship amongst food and human stool *Shigella* isolates [13]. These findings indicate that foods are important vehicle of transmission of MDR *Shigella* to human causing acute intestinal and extraintestinal diseases.

## Conclusions

We detected *Shigella* spp. in 4.84 and 7.7% of food and stool samples respectively. We observed *Sh. sonnei* as the predominant species in both food and stool samples. We observed food isolates resistant to tetracycline and the clinical isolates strongly resistant to imipenem, amikacin and azithromycin. All clinical isolates were susceptible to tetracycline, chloramphenicol and nalidixic acid. 50% and 85.7% of the food and clinical isolates respectively were MDR. We found a close genetic relatedness between the isolates from food and clinical samples. The present study revealed a high prevalence of MDR *Shigella* with close genetic relatedness from food and stool samples indicating poor hygiene practices. Our findings also indicate that MDR *Shigella* can be transmitted through foods and cause infectious disease in human. Performing other studies is of great importance to investigate genetic relatedness between MDR *Shigella* isolates from food and clinical samples.

## Limitations

- 43 *Shigella* isolates from all food and clinical samples are not sufficient to evaluate the genetic relatedness and the antimicrobial susceptibility of the isolates.
- RAPD-PCR method is not adequately precise and reproducible for genotyping of *Shigella* isolates; other sequence based genotyping techniques have been recommended.

## Data Availability

All data used and analyzed at the present study are available from the corresponding author on a reasonable request.

## Abbreviations

HUS: Hemorrhagic Uremic Syndrome
DNA: DeoxyriboNucleic Acid
PCR: Polymerase Chain Reaction
BHI: Bovine Heart Infusion
RAPD: Random Amplification of Polymorphic DNA
MDR: MultiDrug Resistant
UPGMA: Unweighted Pair Group Method with Arithmetic mean
CLSI: Clinical and Laboratory Standards Institute
spp.: Species

## References

[1] Lampel KA, Formal SB, Maurelli AT. A Brief History of Shigella. EcoSal Plus. 2018;8(1):124–59. 10.1128/ecosalplus.esp-0006-2017 PMC8559768 29318984

[2] Puzari M, Sharma M, Chetia P. Emergence of antibiotic resistant Shigella species: a matter of concern. J Infect Public Health. 2018;11(4):451–4. 10.1016/j.jiph.2017.09.025 29066021

[3] Schnupf P, Sansonetti PJ. Shigella pathogenesis: new insights through advanced methodologies. Bacteria Intracell. 2019;1(1):15–39. 10.1128/microbiolspec.bai-0023-2019 PMC11588159 30953429

[4] Njamkepo E, Fawal N, Tran-Dien A, Hawkey J, Strockbine N, Jenkins C (2016). Global phylogeography and evolutionary history of Shigella dysenteriae type 1. Nat Microbiol.

[5] Killackey SA, Sorbara MT, Girardin SE (2016). Cellular aspects of Shigella pathogenesis: focus on the manipulation of host cell processes. Front Cell Infect Microbiol.

[6] Abolghait SK, Fathi AG, Youssef FM, Algammal AM (2020). Methicillin-resistant Staphylococcus aureus (MRSA) isolated from chicken meat and giblets often produces staphylococcal enterotoxin B (SEB) in non-refrigerated raw chicken livers. Int J Food Microbiol.

[7] Algammal AM, El-Kholy AW, Riad EM, Mohamed HE, Elhaig MM, Yousef SAA (2020). Genes encoding the virulence and the antimicrobial resistance in enterotoxigenic and shiga-toxigenic *E. coli* isolated from diarrheic calves. Toxins..

[8] Enany ME, Algammal AM, Nasef SA, Abo-Eillil SA, Bin-Jumah M, Taha AE (2019). The occurrence of the multidrug resistance (mdr) and the prevalence of virulence genes and QACs resistance genes in *E. coli* isolated from environmental and avian sources. AMB Express.

[9] Algammal AM, Mohamed MF, Tawfiek BA, Hozzein WN, El Kazzaz WM, Mabrok M (2020). Molecular typing, antibiogram and PCR-RFLP based detection of Aeromonas hydrophila complex isolated from Oreochromis niloticus. Pathogens.

[10] Algammal AM, Enany ME, El-Tarabili RM, Ghobashy MO, Helmy YA (2020). Prevalence, antimicrobial resistance profiles, virulence and enterotoxin-determinant genes of MRSA isolated from subclinical bovine mastitis samples in Egypt. Pathogens.

[11] Algammal AM, Mabrok M, Sivaramasamy E, Youssef FM, Atwa MH, El-Kholy AW (2020). Emerging MDR-Pseudomonas aeruginosa in fish commonly harbor opr L and tox A virulence genes and bla TEM, bla CTX-M, and tet A antibiotic-resistance genes. Sci Rep.

[12] Keusch GT (2009). Shigellosis. Bacterial infections of humans.

[13] Mokhtari W, Nsaibia S, Majouri D, Ben Hassen A, Gharbi A, Aouni M (2012). Detection and characterization of Shigella species isolated from food and human stool samples in Nabeul, Tunisia, by molecular methods and culture techniques. J Appl Microbiol.

[14] Jian M-J, Perng C-L, Sun J-R, Cheng Y-H, Chung H-Y, Cheng Y-H (2019). Multicentre MDR Elizabethkingia anophelis isolates: Novel random amplified polymorphic DNA with capillary electrophoresis systems to rapid molecular typing compared to genomic epidemiology analysis. Sci Rep.

[15] Kumar NS, Gurusubramanian G (2011). Random amplified polymorphic DNA (RAPD) markers and its applications. Sci Vis.

[16] Marami D, Hailu K, Tolera M (2018). Prevalence and antimicrobial susceptibility pattern of Salmonella and Shigella species among asymptomatic food handlers working in Haramaya University cafeterias, Eastern Ethiopia. BMC Res Notes.

[17] Kassim A, Omuse G, Premji Z, Revathi G (2016). Comparison of Clinical Laboratory Standards Institute and European Committee on Antimicrobial Susceptibility Testing guidelines for the interpretation of antibiotic susceptibility at a University teaching hospital in Nairobi, Kenya: a cross-sectional study. Ann Clin Microbiol Antimicrob.

[18] Berthold-Pluta A, Garbowska M, Stefańska I, Pluta A (2017). Microbiological quality of selected ready-to-eat leaf vegetables, sprouts and non-pasteurized fresh fruit-vegetable juices including the presence of Cronobacter spp. Food Microbiol.

[19] Pavel AB, Vasile CI (2012). PyElph-a software tool for gel images analysis and phylogenetics. BMC Bioinform.

[20] Kotloff KL, Riddle MS, Platts-Mills JA, Pavlinac P, Zaidi AK (2018). Shigellosis. Lancet.

[21] McCrickard LS, Crim SM, Kim S, Bowen A (2018). Disparities in severe shigellosis among adults—Foodborne diseases active surveillance network, 2002–2014. BMC Public Health.

[22] Bakhshi B, Afshari N, Fallah F (2018). Enterobacterial repetitive intergenic consensus (ERIC)-PCR analysis as a reliable evidence for suspected Shigella spp. outbreaks. Braz J Microbiol..

[23] Kahsay AG, Muthupandian S (2016). A review on Sero diversity and antimicrobial resistance patterns of Shigella species in Africa, Asia and South America, 2001–2014. BMC Res Notes.

[24] Nikfar R, Shamsizadeh A, Darbor M, Khaghani S, Moghaddam M (2017). A Study of prevalence of Shigella species and antimicrobial resistance patterns in paediatric medical center, Ahvaz, Iran. Iran J Microbiol.

[25] Zamanlou S, Rezaee MA, Aghazadeh M, Ghotaslou R, Nave HH, Khalili Y (2018). Genotypic diversity of multidrug resistant Shigella species from Iran. Infection & chemotherapy.

[26] Ranjbar R, Farahani A (2019). Shigella: antibiotic-resistance mechanisms and new horizons for treatment. Infect Drug Resist.

[27] Ranjbar R, Behnood V, Memariani H, Najafi A, Moghbeli M, Mammina C (2016). Molecular characterisation of quinolone-resistant Shigella strains isolated in Tehran, Iran. J Glob Antimicrob Resist.

[28] Shahin K, Bouzari M, Wang R, Yazdi M (2019). Prevalence and molecular characterization of multidrug-resistant Shigella species of food origins and their inactivation by specific lytic bacteriophages. Int J Food Microbiol.

[29] Aklilu A, Kahase D, Dessalegn M, Tarekegn N, Gebremichael S, Zenebe S (2015). Prevalence of intestinal parasites, salmonella and shigella among apparently health food handlers of Addis Ababa University student’s cafeteria, Addis Ababa, Ethiopia. BMC Res Notes.

